# Skin Longevity Medicine: A Hierarchical Framework for Personalized Skin Health and Aging Intervention

**DOI:** 10.7759/cureus.117987

**Published:** 2026-10-15

**Authors:** Liangsen Zhao, Yong Liao

**Affiliations:** 1 Department of Medicine, Bloomage Biotechnology Corporation Limited, Beijing, CHN

**Keywords:** aging, extracellular matrix, multimodal intervention, personalized medicine, precision health, skin longevity

## Abstract

The expanding framework of aging hallmarks and the emerging paradigm of precision geromedicine have created new opportunities for translating molecular geroscience into clinical skin health. Skin longevity-defined as the sustained maintenance of tissue function, structural integrity, and biological resilience-represents a conceptual shift from appearance-oriented anti-aging toward evidence-based, multimodal intervention. This review examines the scientific basis of skin longevity, including the cutaneous manifestations of aging hallmarks, dermal fibroblast senescence, and extracellular matrix (ECM) degeneration, while identifying critical limitations in current fragmented intervention approaches. We propose a hierarchical intervention pyramid comprising five pillars: systemic metabolic and nutritional optimization, lifestyle and psychoneurobiological regulation, epidermal barrier maintenance and local epigenetic modulation, ECM homeostasis and regenerative injectables, and energy-based devices. This framework emphasizes temporal sequencing, biological synergy, and individualized precision strategies rather than isolated treatment accumulation. By integrating evidence from nutraceuticals, topical therapeutics, biostimulatory agents, and procedural interventions within a unified mechanistic hierarchy, this perspective aims to stimulate scholarly discourse and advance the development of personalized skin longevity medicine as a component of precision health.

## Introduction and background

In 2023, López-Otín et al. expanded the hallmarks of aging from nine to 12, and in 2025, Kroemer et al. further advanced the framework of "precision geromedicine" by introducing two additional hallmarks: extracellular matrix (ECM) alterations and psychosocial isolation [1,2]. This paradigm shift is particularly evident in dermatology. The skin is not only the most visible indicator of systemic aging but also the organ with the highest ECM content and the broadest range of available interventions [3,4]. The concepts of skin longevity and Skinspan™ were formally introduced between 2024 and 2025 and are defined as the duration over which the skin maintains optimal structural integrity and physiological function [3,4]. More specifically, skin longevity encompasses the sustained preservation of epidermal barrier function, dermal ECM homeostasis, fibroblast functional activity, tissue regenerative capacity, and cutaneous immune competence. Beyond mere structural integrity and aesthetic appearance, the core objective of skin longevity is to extend the functional lifespan of cutaneous tissue units-delaying the transition from healthy, functionally active cells and tissues to senescent, dysfunctional states-thereby prolonging the period during which skin maintains its full biological capacity rather than merely slowing visible decline. Unlike traditional anti-aging approaches that primarily focus on aesthetic improvement, the skin longevity framework emphasizes the long-term preservation of tissue structure and biological integrity.

Nevertheless, a substantial gap remains between current skin rejuvenation practices and the scientific objectives of skin longevity. Existing interventions are largely fragmented: topical formulations primarily target the superficial epidermis, energy-based devices rely on controlled tissue injury, injectable fillers provide volumization and tissue remodeling, and oral supplements are often based on single-ingredient paradigms, lacking a unified theoretical framework and coordinated temporal design [5]. From a regulatory perspective, most injectable dermal fillers and biostimulatory agents are classified as Class III medical devices [6], whereas their mechanisms of action differ considerably from those of regenerative medicine and energy-based interventions. Consequently, categorization based solely on intervention type inevitably results in conceptual overlap and hierarchical inconsistency. This review aims to evaluate the current landscape and limitations of the field; propose a multimodal integrative framework based on intervention dimensions, delivery pathways, and biological mechanisms; and discuss future directions toward precision-based approaches. To inform this analysis, a comprehensive literature search was conducted across PubMed/MEDLINE, Web of Science, Scopus, and Google Scholar, covering publications from January 2020 to August 2026, with priority given to studies published within the last five years. Key search terms included combinations of "skin longevity," "skin aging," "skinspan," "extracellular matrix," "fibroblast senescence," "dermal regeneration," "biostimulatory agents," "energy-based devices," "nutrigenomics," and "precision dermatology." Inclusion criteria prioritized peer-reviewed original research articles, randomized controlled trials, systematic reviews, and mechanistic studies in English, with a focus on human clinical evidence and translational research. Seminal articles and landmark references outside the search period were also included for foundational context.

## Review

Scientific basis and clinical needs of skin longevity

Skin Mapping of Aging Hallmarks and ECM Degeneration

All 14 hallmarks of aging exhibit distinct manifestations within skin tissue [2,7,8]. For example, genomic instability is reflected by the accumulation of ultraviolet (UV)-induced DNA photodamage products, whereas epigenetic alterations can be quantified using the Horvath skin clock. Energy-based treatments have also been shown to partially reverse age-associated DNA methylation changes [9]. Cellular senescence is characterized by the accumulation of p16INK4a-positive dermal fibroblasts, which secrete senescence-associated secretory phenotype (SASP) factors, including IL-6, TNF-α, and matrix metalloproteinases (MMPs), thereby establishing a chronic pro-inflammatory microenvironment [8]. Single-cell transcriptomic studies further reveal distinct senescence-driving cell populations within the epidermis and dermis [10].

ECM degeneration constitutes the fundamental structural basis of skin aging. Type I and type III collagen account for approximately 80%-85% of total skin protein, and their relative proportions change substantially with age. Progressive depletion of type III collagen directly contributes to reduced skin elasticity and impaired wound healing [11]. Because ECM proteins possess exceptionally long half-lives-for example, approximately 15 years for type I collagen-accumulated damage is difficult to eliminate through natural turnover [11]. A 2026 study published in Science demonstrated that ECM composition and mechanical properties are critical determinants of regeneration versus fibrosis. Stabilization of the hyaluronic acid (HA) network through HAPLN1 was shown to induce bone regeneration in injuries that would otherwise result in permanent scarring, redefining the ECM from a passive structural scaffold to an active regulator of signaling processes [12].

Fibroblast Senescence and the SASP Cascade

Dermal fibroblasts are the principal producers of ECM and the central driver cells of skin aging. As long-lived, low-proliferative post-mitotic cells, fibroblasts are unable to eliminate accumulated exogenous damage through cellular turnover [8]. Recent single-cell studies have identified two additional features of fibroblast senescence: "identity noise," characterized by blurred boundaries between functional fibroblast subsets, and "adipogenic acquisition," whereby senescent fibroblasts acquire PPARγ-mediated adipogenic traits under systemic metabolic regulation [10]. The detrimental effects of fibroblast senescence extend beyond intrinsic functional decline. Through SASP-mediated paracrine signaling, senescent fibroblasts disrupt keratinocyte differentiation, alter melanocyte homeostasis, suppress immune surveillance, and establish a self-propagating cycle of "senescent cells → SASP → neighboring cell senescence" [8].

Unmet Clinical Needs: From Aesthetic Scores to Functional Endpoints

Current assessment systems for skin rejuvenation remain limited. Most clinical studies rely primarily on visual improvements or subjective outcomes, such as wrinkle severity scores, patient satisfaction, and the Global Aesthetic Improvement Scale (GAIS), while objective measurements of biological skin age, ECM mechanical properties, barrier function, and regenerative capacity are rarely incorporated [5]. The skin longevity framework calls for a transition from appearance-based metrics to functional tissue-level endpoints. Clinically applicable assessments currently include transepidermal water loss (TEWL), skin elastic modulus, and dermal thickness measured by high-frequency ultrasound; however, none can comprehensively reflect biological age or regenerative potential [4,13]. Epigenetic clocks offer a promising tool for objective evaluation, although their application remains largely confined to research settings.

Current interventions: status and limitations

Topical Formulations

Topical formulations constitute the foundation of skin longevity management. Core components include broad-spectrum sunscreens, moisturizers, topical retinoids, antioxidants, and barrier-repair agents. Daily application of a sunscreen with sun protection factor (SPF) 10/persistent pigment darkening (PPD) 17.5 for one year was shown by in vivo three-dimensional multiphoton microscopy to prevent seasonal changes in melanin, elastin, and the skin aging index (second harmonic generation-to-autofluorescence aging index of dermis (SAAID)), providing direct histological evidence supporting photoprotection as a cornerstone of skin longevity [14]. Topical retinoids (0.025%-0.1%) promote collagen synthesis, inhibit MMPs, and improve dermal-epidermal junction (DEJ) architecture through activation of RAR/RXR signaling [4].

The emerging concept of "longevity cosmeceuticals" requires products to directly target skin aging hallmarks and demonstrate efficacy through clinical validation [15]. In the area of mitochondrial protection, ergothioneine exerts targeted antioxidant activity via the OCTN1 transporter; ectoine functions as a compatible solute that stabilizes cellular membranes and protein structures; and salidroside mitigates UV-induced keratinocyte injury by inducing SIRT1-dependent autophagy [16,17]. Nevertheless, topical interventions face an inherent limitation: the stratum corneum barrier restricts the penetration depth of most active ingredients, limiting their bioavailability within the dermis and their ability to exert effects on deeper dermal structures [18].

Injectable Interventions and Regenerative Medicine

Injectable therapies represent the most direct approach for targeting the ECM microenvironment. HA fillers remain the most widely used products, with recent advances including bioorthogonal crosslinking and thiol-mediated self-crosslinking technologies designed to reduce crosslinker-associated toxicity and improve tissue integration and persistence [5]. Biostimulatory injectables, which promote endogenous collagen synthesis, have become one of the fastest-growing categories in aesthetic medicine. As an amorphous polymer, poly-D,L-lactic acid (PDLLA) exhibits a more uniform degradation profile and a lower risk of granuloma formation than crystalline poly-L-lactic acid (PLLA). A randomized, evaluator-blinded, multicenter study involving 260 participants demonstrated that PDLLA was non-inferior to HA fillers for nasolabial fold correction in terms of efficacy and safety, while its collagen-stimulatory effects persisted for more than 18 months [19]. Mechanistically, PDLLA-derived lactate promotes collagen synthesis through activation of the hydroxycarboxylic acid receptor 2 (HCA2) [5].

The three pillars of regenerative medicine-cell-based therapies, biochemical signaling modulation, and biostimulatory scaffolds-have all been explored in dermatology. However, most regenerative approaches remain supported by relatively low levels of evidence and are hindered by the lack of standardized manufacturing protocols and long-term safety data. Quality control concerns are particularly prominent for exosome-based products [20].

Energy-Based Devices

Energy-based devices initiate tissue repair through controlled thermal or mechanical injury. Mechanistic studies have shown that laser-induced mechanical tension activates fibroblasts through the YAP/TAZ signaling pathway and remodels chromatin architecture, thereby partially reversing age-associated epigenetic alterations [9]. However, clinical outcomes remain highly operator-dependent, and the optimal sequencing and dose matching of combination protocols require validation through prospective studies.

A common limitation shared by current interventions is the absence of an integrated hierarchical framework based on delivery pathways and biological mechanisms. Instead, individual modalities are typically categorized according to product or device type. In clinical practice, treatment often begins directly with energy-based procedures or localized volumization. However, when patients present with elevated cortisol levels, chronic low-grade inflammation, or impaired barrier function, wound-healing responses may be compromised, potentially leading to post-inflammatory hyperpigmentation or accelerated MMP-mediated matrix degradation. This highlights a fundamental clinical challenge: interventions applied in the absence of an optimized biological foundation may fail to achieve their intended outcomes.

Multimodal integrated strategies for skin longevity medicine

Hierarchical Logic of the Intervention Pyramid

Based on the preceding analysis, we propose that the central challenge in skin longevity medicine is not the discovery of new individual targets, but the systematic integration of existing interventions according to the principles of hierarchical progression, homeostasis prioritization, and temporal synergy. We therefore propose a multimodal intervention pyramid for skin longevity, consisting of four hierarchical levels and five core pillars, arranged from the foundation to the apex (Figures 1, 2). The hierarchical positioning of interventions within this framework is determined by three interrelated criteria: (1) the depth and breadth of biological action across systemic, barrier, microenvironmental, and structural levels; (2) the strength and consistency of supporting clinical evidence, ranging from robust randomized controlled trial data for established interventions to limited clinical validation for investigational approaches; and (3) the logical temporal sequence of clinical application to ensure adequate biological preparation. Figure 1 presents a concentric circle diagram illustrating the five core pillars of skin longevity medicine, with representative intervention modalities listed for each pillar; the listed modalities constitute common examples and are not intended to be exhaustive, and the spatial proximity of items within each sector reflects conceptual relatedness rather than hierarchical ranking. Figure 2 depicts a four-tiered pyramid representing the proposed hierarchical logic and temporal sequencing of multimodal interventions, progressing from foundational systemic approaches at the base to energy-based technologies at the apex; this hierarchical structure is derived from a synthesis of the current literature on intervention mechanisms, clinical evidence, and biological preparation principles. It should be emphasized that while the individual interventions discussed are supported by varying levels of clinical and mechanistic evidence, the integrated hierarchical framework itself represents the authors' conceptual synthesis based on the current literature, and the proposed temporal sequencing requires prospective clinical validation.

**Figure 1:**
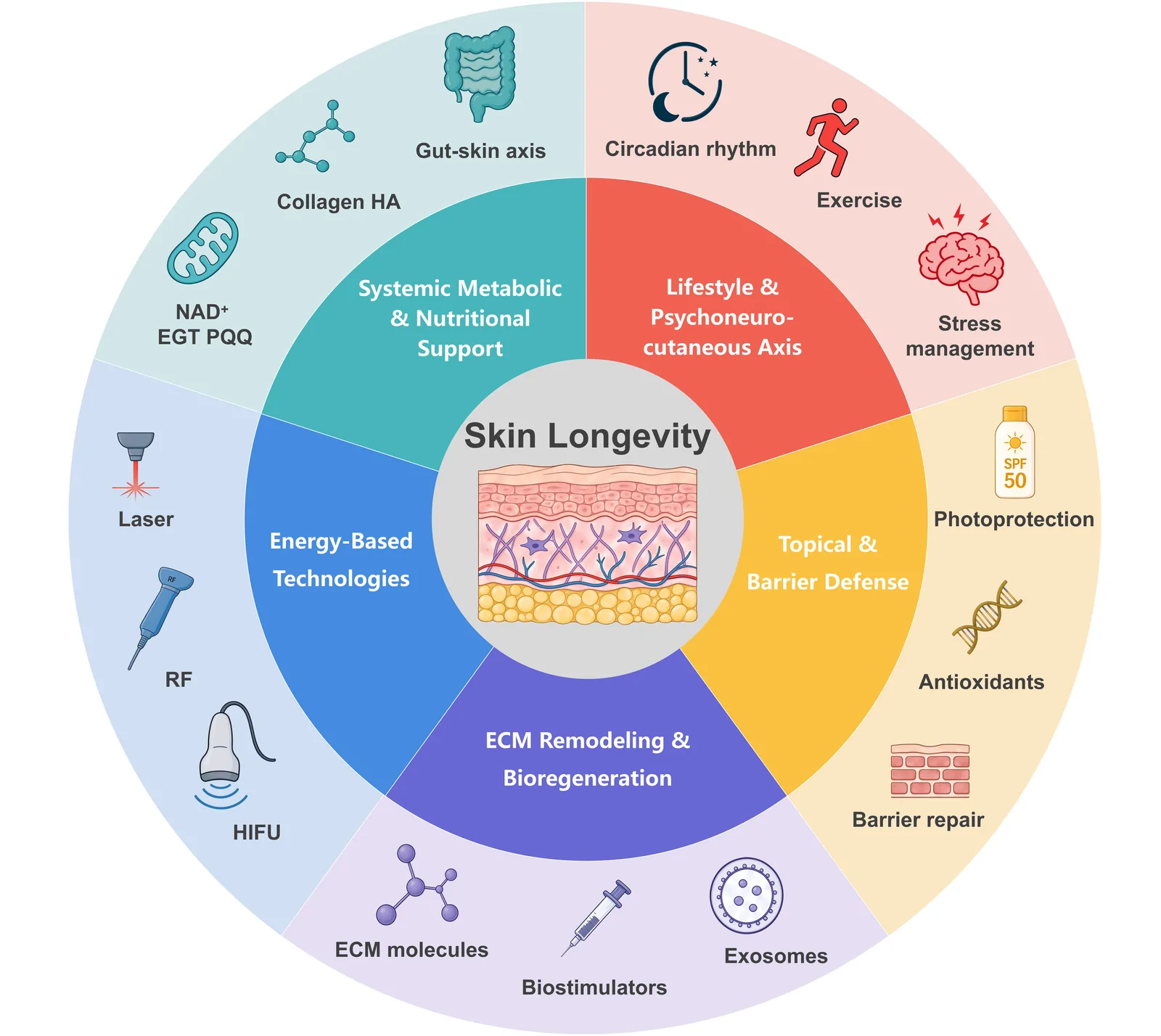
Five Pillars of Skin Longevity Medicine. This concentric circle diagram summarizes the five intervention domains of a multimodal skin longevity framework, with representative intervention modalities listed for each pillar (not exhaustive). This figure provides an overview of the scope of intervention categories, independent of their hierarchical positioning or temporal sequencing. HA: hyaluronic acid; NAD+: nicotinamide adenine dinucleotide; EGT: ergothioneine; PQQ: pyrroloquinoline quinone; ECM: extracellular matrix; RF: radiofrequency; HIFU: high-intensity focused ultrasound Original figure created by the authors using PowerPoint (Microsoft Corp., Redmond, WA, USA) and iconfont (Alibaba Group, Hangzhou, Zhejiang, China).

**Figure 2:**
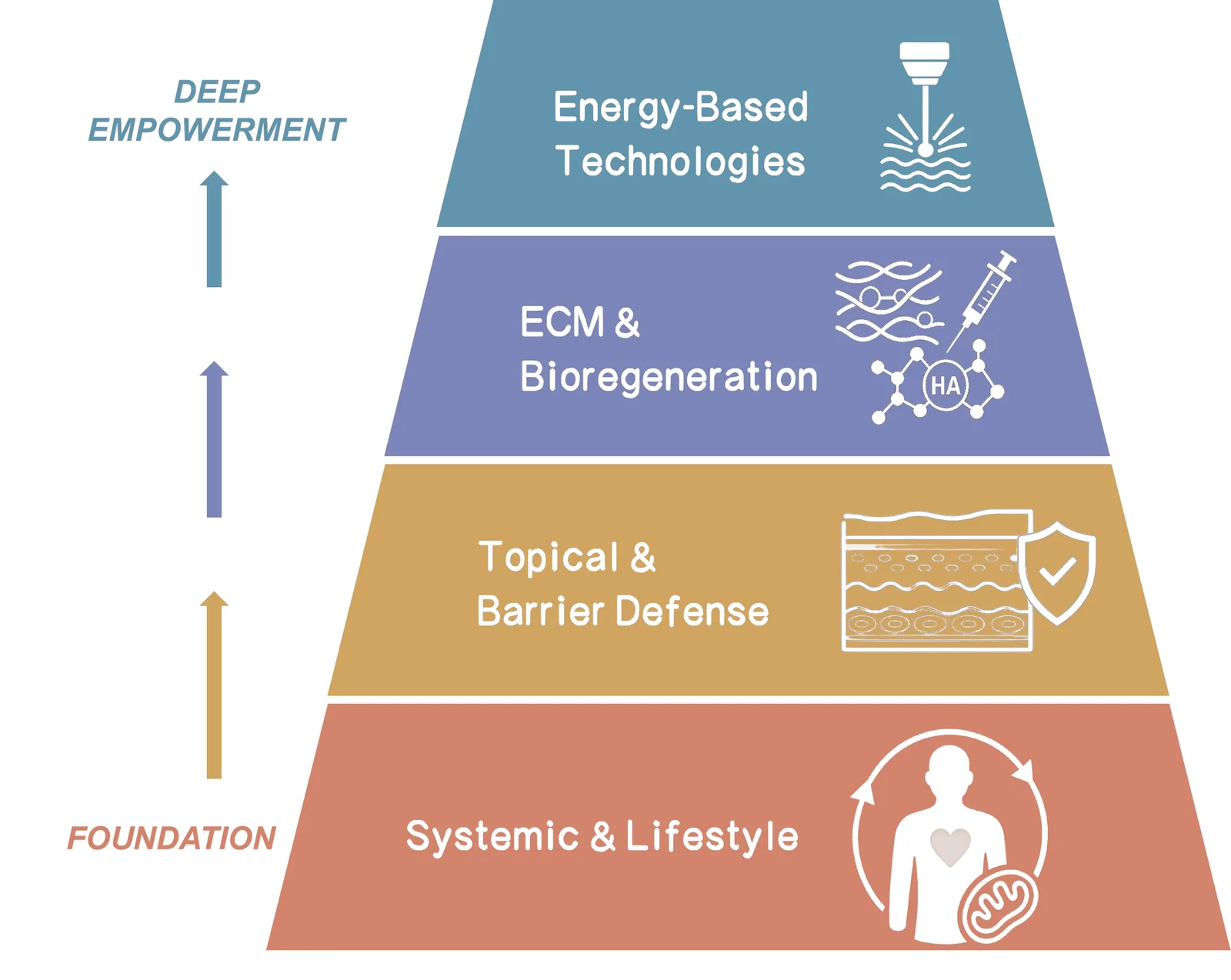
Multimodal Intervention Pyramid for Skin Longevity. This pyramid diagram represents the proposed hierarchical organization and temporal sequencing of multimodal skin longevity interventions, progressing from foundational systemic approaches at the base (to be implemented first) to energy-based technologies at the apex (to be applied after adequate biological preparation). In contrast to Figure 1, which summarizes the five intervention domains, this figure illustrates their logical ordering and clinical application sequence. ECM: extracellular matrix Original figure created by the authors using PowerPoint (Microsoft Corp., Redmond, WA, USA) and iconfont (Alibaba Group, Hangzhou, Zhejiang, China).

The hierarchy follows a progression from systemic foundations to barrier protection, microenvironment remodeling, and finally physical and structural rejuvenation. The base layer comprises two pillars-systemic metabolic and nutritional interventions, and psychobehavioral-lifestyle interventions-which regulate endogenous homeostasis and inflammatory status. The second tier consists of epidermal barrier maintenance and local epigenetic modulation, providing protection against exogenous insults. The third tier, ECM homeostasis and regenerative injectables, represents the core remodeling layer of the pyramid. This level directly replenishes depleted HA and collagen, restores fibroblast mechanical tension, and promotes sustained ECM renewal and microvascular regeneration through biostimulatory interventions. At the apex, physical and energy-based devices provide macroscopic tissue tightening and deep mechanotransductive stimulation. The evidence base, proposed mechanisms, principal clinical endpoints, and major limitations of representative interventions across all five hierarchical levels are systematically summarized in Table 1. It should be noted that the references cited in Table 1 constitute a selected subset of the supporting literature included in this review, and that additional relevant evidence exists in the broader public domain beyond the scope of the cited references. This hierarchical framework addresses the longstanding challenge of inadequate biological preparation in aesthetic medicine and establishes ECM remodeling and regenerative interventions as the critical bridge between routine skin maintenance and energy-based procedures.

**Table 1:** Summary of Skin Longevity Interventions: Mechanisms, Evidence Levels, Clinical Endpoints, and Limitations. ECM: extracellular matrix; RCT: randomized controlled trial; HA: hyaluronic acid; TEWL: transepidermal water loss; NMN: nicotinamide mononucleotide; UV: ultraviolet; PQQ: pyrroloquinoline quinone; HPA: hypothalamic-pituitary-adrenal; SPF: sun protection factor; MMP: matrix metalloproteinase; SAAID: second harmonic generation-to-autofluorescence aging index of dermis; DEJ: dermal-epidermal junction; ROS: reactive oxygen species; PLLA: poly-L-lactic acid; PN: polynucleotide; PDRN: polydeoxyribonucleotide; PIH: post-inflammatory hyperpigmentation; RF: radiofrequency; HIFU: high-intensity focused ultrasound; SMAS: superficial musculoaponeurotic system; IPL: intense pulsed light; PDL: pulsed dye laser; Nd:YAG: neodymium-doped yttrium aluminum garnet

Pillar	Intervention	Proposed mechanism	Evidence level	Principal clinical endpoint	Major limitations	Refs
I. Systemic metabolic & nutritional	Hydrolyzed collagen peptides (5-10 g/day)	Provides ECM precursors (glycine, proline, hydroxyproline); stimulates fibroblast collagen synthesis	RCT, meta-analysis	Skin hydration, elasticity, overall skin quality	Optimal dosage and long-term effects not fully established; formulation variability	[21,22]
	Oral hyaluronic acid (120 mg/day)	Improves tissue hydration; modulates CD44/RHAMM signaling; stimulates endogenous HA synthesis	RCT, meta-analysis	Skin hydration, TEWL reduction, elasticity, wrinkle severity, dermal density	Molecular weight-dependent efficacy; long-term safety data limited	[23,24]
	NMN (NAD+ precursor)	Replenishes cellular NAD+; activates sirtuins; attenuates UVB-induced epidermal damage	Preclinical + human pilot	Skin hydration, elasticity, UV-induced immunosuppression	Large-scale RCTs lacking; optimal dosage undefined	[25]
	PQQ (20 mg/day)	Promotes mitochondrial biogenesis via PGC-1α activation	Clinical study	Forearm TEWL reduction	Limited clinical data; skin-specific outcomes understudied	[26]
	Ergothioneine (25 mg/day)	Targeted antioxidant via OCTN1 transporter; anti-inflammatory effects	RCT	Skin moisture, wrinkle/texture scores, pigmentation	Long-term efficacy data limited; formulation-dependent bioavailability	[27]
	Dietary patterns (Okinawan, Nordic, Blue Zone)	High polyphenol/omega-3 intake; regulates NF-κB, mTOR, autophagy pathways	Epidemiological + mechanistic	Systemic inflammatory markers, skin health correlates	Difficult to isolate skin-specific effects; adherence challenges	[28]
II. Lifestyle & psychoneurobiological	Exercise (aerobic + resistance training)	Irisin secretion; AMPK/PGC-1α pathway; reduces inflammatory mediators; upregulates ECM gene expression	RCT (16-week, n = 61)	Skin elasticity, dermal thickness, upper dermal structure	Optimal frequency/intensity for skin outcomes not standardized; limited sample size	[29]
	Sleep & circadian rhythm maintenance	Epidermal circadian clock regulates barrier function, cell proliferation, DNA repair	Mechanistic + clinical	Barrier function, skin recovery	Difficult to quantify optimal sleep parameters for skin outcomes	[30]
	Stress management & social engagement	Reduces HPA axis cortisol elevation; preserves filaggrin and HA production; mitigates psychosocial isolation	Mechanistic + epidemiological	Barrier function, inflammatory markers	Subjective outcomes; lack of standardized interventions	[2,31,32]
III. Epidermal barrier & topical	Broad-spectrum photoprotection (SPF ≥ 30)	Prevents UV-induced DNA photodamage; inhibits MMP activation	In vivo 3D multiphoton microscopy (1-year)	Melanin, elastin, SAAID index	Adherence challenges; UVA protection variability	[14]
	Topical retinoids (0.025%-0.1%)	RAR/RXR signaling; collagen synthesis; MMP inhibition; DEJ architecture improvement	Network meta-analysis (23 RCTs, n = 3,905)	Fine wrinkles, pigmentation, skin texture	Irritation; teratogenicity risk; photosensitivity	[33]
	Topical antioxidants & barrier repair (Vit C/E, ferulic acid, ceramides)	Neutralizes ROS; restores lipid bilayer; reduces TEWL	Clinical studies	Photoprotection, TEWL, skin hydration	Stability issues; penetration depth limited; effect mainly superficial	[4]
	Longevity cosmeceuticals (ectoine, salidroside, ergothioneine)	Mitochondrial protection; SIRT1-dependent autophagy; membrane stabilization; OCTN1-mediated antioxidant	Preclinical + early clinical	Antioxidant capacity, barrier function, UV protection	Limited large-scale clinical validation; penetration depth restricted	[15-17]
IV. ECM homeostasis & regenerative injectables	HA fillers (crosslinked/non-crosslinked)	Restores volume; CD44/RHAMM signaling; tissue hydration; fibroblast migration; ECM synthesis	RCT, clinical experience	Volume restoration, skin hydration, nasolabial fold correction	Duration limited; vascular occlusion risk; immune reactions rare	[34]
	PDLLA (poly-D,L-lactic acid)	Lactate-mediated HCA2 receptor activation; neocollagenesis; biostimulatory scaffold	RCT (260 participants, multicenter, evaluator-blinded)	Nasolabial fold correction; collagen stimulation persisting >18 months	Gradual onset; requires multiple sessions; nodule risk (lower than PLLA)	[19]
	PN/PDRN (animal-derived)	Adenosine A2A receptor activation; anti-inflammatory; scaffold support + biochemical signaling	Clinical studies	Skin hydration, texture improvement, regeneration	Standardization variability; long-term safety data limited	[19]
	Calcium hydroxylapatite (CaHA)	Biostimulatory scaffold; induces collagen and elastin synthesis	Clinical studies + RCT	Volume restoration, skin laxity improvement	Higher viscosity; difficult to reverse; nodule risk	[5]
	Exosomes/extracellular vesicles	Paracrine signaling; growth factor delivery; immunomodulation	Preclinical + early clinical	Skin rejuvenation, wound healing (preliminary)	Lack of standardized manufacturing; quality control weak; long-term safety unknown; regulatory status unclear	[20]
V. Energy-based devices	Fractional lasers (non-ablative, CO₂)	Controlled thermal injury; collagen remodeling; YAP/TAZ mechanotransduction; upregulates collagen genes	Clinical studies + histological evidence	Wrinkle improvement, type III collagen increase, chromatin remodeling	Operator-dependent; downtime; PIH risk in darker skin types	[9,35]
	Tissue tightening devices (RF, HIFU)	Deep tissue heating; collagen contraction; neocollagenesis; SMAS layer thermal coagulation	Clinical studies	Skin laxity, tissue tightening, brow lift, jawline definition	Pain; variable efficacy; nerve injury risk (HIFU); multiple sessions required	[4,5]
	Pigment & vascular lasers (IPL, Q-switched, picosecond, PDL, Nd:YAG)	Selective photothermolysis; pigment particle fragmentation; vascular targeting (hemoglobin absorption)	Clinical studies	Pigmentary lesion clearance, telangiectasia, persistent erythema, skin texture	PIH risk; multiple sessions; less precise (IPL); purpura (PDL); not suitable for all skin types	[4,5]

Pillar I: Systemic Metabolic and Nutritional Interventions

Systemic interventions influence skin health through circulating metabolic pathways and the gut-brain-skin axis, serving as the foundational component of multimodal skin longevity strategies [31,32]. With respect to ECM synthetic precursors, multiple randomized controlled trials have demonstrated that hydrolyzed collagen peptides (5-10 g/day) improve skin hydration, elasticity, and overall skin quality [21]. A collagen amino acid formulation containing glycine, proline, and hydroxyproline in a 3:1:1 ratio extended lifespan by 6%-27% in Caenorhabditis elegans and reduced biological age by approximately 1.4 years after six months of supplementation in humans (p = 0.04) [22]. For oral HA, a randomized, double-blind, placebo-controlled trial involving 150 participants demonstrated that daily supplementation with 120 mg high-molecular-weight sodium hyaluronate (1.8 MDa) for 12 weeks significantly improved skin hydration, reduced TEWL, enhanced elasticity, decreased wrinkle severity, and increased both epidermal thickness and dermal density [23]. Meta-analyses of multiple randomized controlled trials have similarly confirmed the beneficial effects of oral HA on skin hydration and elasticity [24].

Among cellular bioenergetic modulators, nicotinamide mononucleotide (NMN), a precursor of NAD+, has been shown to attenuate UVB-induced epidermal thickening and wrinkle formation while improving skin hydration and elasticity. Human studies further indicate that oral NAD+ precursors increase circulating NAD+ levels and reduce UV-induced cutaneous immunosuppression [25]. Pyrroloquinoline quinone (PQQ) promotes mitochondrial biogenesis through activation of PGC-1α, and supplementation at 20 mg/day for eight weeks has been reported to attenuate increases in forearm TEWL [26]. Ergothioneine (25 mg/day) exerts antioxidant and anti-inflammatory effects through OCTN1-mediated cellular uptake. In a 12-week randomized, double-blind, placebo-controlled trial involving 80 healthy women, oral supplementation with an ergothioneine-rich Pleurotus extract (25 mg/day) significantly improved skin moisture content at eight weeks and wrinkle/texture scores at 12 weeks; plasma ergothioneine concentrations increased 4.7-fold and correlated with improvements in skin hydration and TEWL [27]. Gut microbiome modulators contribute to intestinal barrier integrity, while short-chain fatty acids suppress NF-κB signaling through FFAR2/3, GPR109a, and PPARγ pathways, thereby mitigating systemic inflammation [32]. From a dietary perspective, Okinawan, Nordic, and Blue Zone dietary patterns share common characteristics, including high polyphenol intake, abundant omega-3 fatty acids, and fermented foods, which exert protective effects on the skin through regulation of NF-κB, mTOR, and autophagy-related pathways [28].

Pillar II: Lifestyle Behaviors and the Psychoneurobiological Axis

Lifestyle interventions represent the most fundamental component of skin longevity management and offer the greatest safety profile at the lowest cost. Regular aerobic exercise promotes mitochondrial biogenesis in fibroblasts through the secretion of irisin and subsequent activation of the AMPK/PGC-1α pathway [4]. A 16-week randomized controlled trial involving 61 middle-aged women demonstrated that both aerobic and resistance exercise significantly improved skin elasticity and upper dermal structure. Resistance training additionally increased dermal thickness, potentially through reductions in circulating inflammatory mediators and upregulation of ECM-related gene expression within the dermis [29]. Adequate sleep and maintenance of circadian rhythms are essential for skin homeostasis. The epidermal circadian clock not only responds to signals from the central nervous system but can also integrate and override neural inputs to preserve cutaneous homeostasis. Tissue-specific reconstruction of Bmal1 has further revealed the gating function of peripheral clocks in regulating skin physiology [30].

In 2025, psychosocial isolation was formally recognized as the 14th hallmark of aging [2]. Chronic psychological stress elevates cortisol levels through activation of the hypothalamic-pituitary-adrenal (HPA) axis, suppressing keratinocyte production of filaggrin and HA. Social isolation and loneliness similarly accelerate aging through persistent neuroendocrine stress responses [2,31]. Accordingly, stress management, social engagement, and emotional regulation should be considered integral components of comprehensive skin longevity strategies.

Pillar III: Epidermal Barrier Maintenance and Local Epigenetic Modulation

Topical interventions serve as a continuous 24/7 protective layer within skin longevity management and should follow a circadian-based strategy of "morning protection and evening repair." Morning regimens should prioritize broad-spectrum photoprotection (SPF ≥ 30, PA++++) combined with antioxidants, such as 10%-20% L-ascorbic acid, vitamin E, and ferulic acid. Evening regimens should emphasize retinoids (0.025%-0.1%) in combination with barrier-repair agents, including ceramides, cholesterol, and free fatty acids [4]. A 2025 network meta-analysis encompassing 23 randomized controlled trials and 3,905 participants demonstrated that isotretinoin, retinol, and tretinoin all significantly improved fine wrinkles, with tretinoin showing the most favorable performance in pigmentation improvement while maintaining the best safety profile [33].

The gating function of the epidermal circadian clock suggests that the timing of topical application is not merely empirical but is supported by circadian biology [30]. The emerging concept of "skin activation" reflects the evolution of topical interventions from conventional moisturization and antioxidation toward the activation of cellular function. The objective is not to prolong cellular lifespan per se, but to maintain healthy, functional cells and optimize tissue renewal dynamics. Within the scientific framework of longevity cosmeceuticals, products should not only improve visible appearance but also directly target hallmarks of skin aging and demonstrate efficacy through clinical validation [15]. This principle should serve as a central criterion for both product development and clinical decision-making.

Pillar IV: ECM Homeostasis and Regenerative Injectables

ECM remodeling and regenerative injectables constitute the central pillar of the multimodal skin longevity pyramid, positioned between routine external maintenance and energy-based interventions. Their primary role is to restore the physical and biochemical microenvironment required for fibroblast function, thereby providing adequate hydration substrates and regenerative capacity for subsequent procedures. For maintenance of matrix homeostasis and biomechanical support, crosslinked and non-crosslinked HA formulations, as well as collagen-based products, represent the principal therapeutic modalities. Non-crosslinked or micro-crosslinked HA, polynucleotides (PNs), and skin booster injections exert microenvironment-modulating effects through interactions with CD44 and RHAMM receptors, regulation of tissue hydration, promotion of fibroblast migration, and stimulation of ECM synthesis [34]. A 2026 Science study suggested that future regenerative strategies should shift from a cell-centered paradigm toward matrix engineering. By optimizing the stability and mechanical properties of HA networks, it may be possible to establish a regenerative microenvironment that favors tissue repair rather than fibrosis [12]. Modulation of ECM stiffness is therefore likely to become a key direction in the development of next-generation injectable therapies [10,12].

Among biostimulatory agents and cell-derived products, major categories include PDLLA, calcium hydroxylapatite (CaHA), exosomes/extracellular vesicles, and PN/polydeoxyribonucleotide (PDRN). PDLLA and CaHA function as biostimulatory scaffolds that induce neocollagenesis. PDLLA promotes collagen synthesis through lactate-mediated activation of HCA2 receptors, with clinical effects persisting for more than 18 months [19]. PN and PDRN, predominantly animal-derived, provide both scaffold support and biochemical signaling, exerting anti-inflammatory and regenerative effects through activation of adenosine A2A receptors [5,19].

In contrast to the established biostimulatory agents discussed above-including HA fillers, PDLLA, CaHA, and PN/PDRN, which are supported by randomized controlled trials or consistent clinical evidence and have defined regulatory pathways-exosome-based products represent an investigational regenerative approach with heterogeneous and rapidly evolving evidence. These products may be derived from adipose-derived stem cells, umbilical cord tissue, or plant sources, whereas Hybrosomes remain at the preclinical stage [5]. From a regulatory perspective, exosome-based products face significant uncertainties: manufacturing standardization remains inadequate, with substantial variability in isolation methods, characterization protocols, and potency assays across producers; long-term safety data are lacking; and the quality of clinical evidence remains generally low, with most studies being small, non-randomized, or preclinical in nature [20]. Clinical application of these products should therefore clearly distinguish between established collagen-stimulatory effects supported by clinical evidence and unverified regenerative medicine claims, and particular caution should be exercised given the current gaps in regulatory oversight, manufacturing consistency, and long-term safety evaluation [20].

Pillar V: Energy-Based Devices

Energy-based devices occupy the apex of the intervention pyramid, initiating tissue repair through controlled injury while providing macroscopic tissue tightening and deep mechanotransductive stimulation. Device selection should be guided by skin phenotype assessment. For predominantly photoaged skin, non-ablative fractional lasers are preferred. For laxity and tissue descent, radiofrequency and high-intensity focused ultrasound are generally favored. For pigmentary disorders, Q-switched lasers, picosecond lasers, or intense pulsed light are appropriate options. For telangiectasia and persistent erythema, pulsed dye lasers (PDL) or long-pulsed neodymium-doped yttrium aluminum garnet (Nd:YAG) lasers are recommended [4,5].

Laser-induced mechanical tension activates fibroblasts through the YAP/TAZ pathway and remodels chromatin architecture, thereby partially reversing age-associated epigenetic alterations [9]. In addition, non-ablative fractional 1,540 nm lasers have been shown to upregulate collagen-related genes, enhance mitochondrial activity, and significantly increase type III collagen expression, providing histological evidence for the ECM-remodeling capacity of energy-based interventions [35]. Importantly, energy-based procedures should be performed only after adequate preparation of the biological foundation and core remodeling layers. In individuals with persistent inflammation or insufficient dermal regenerative capacity, wound-healing responses may be impaired, increasing the risk of adverse outcomes. Although the combination of energy-based devices and biostimulatory agents is theoretically synergistic, the optimal treatment intervals and dosing strategies remain to be established through prospective clinical studies [5,9].

Temporal Sequencing and Synergy of Multimodal Interventions

The primary value of multimodal interventions lies in biological synergy rather than simple treatment accumulation. An integrated strategy combining topical and systemic interventions has already been proposed, whereby topical retinoids and antioxidants are paired with oral NAD+ precursors, collagen peptides, and microbiome-modulating agents to achieve complementary benefits [32].

As a proposed conceptual framework derived from the synthesis of current mechanistic and clinical evidence rather than from prospective comparative trials, treatment sequencing should follow the hierarchical logic of the intervention pyramid. It should be noted that while each individual intervention within this sequence is supported by varying levels of clinical evidence (as summarized in Table 1), direct prospective evidence validating the optimal order and timing of this specific multimodal sequence remains limited. Systemic metabolic optimization and lifestyle modification should be implemented first as a foundational preconditioning phase. This should be followed by establishment of epidermal barrier protection, then ECM microenvironment remodeling and regenerative injectable therapies, and finally energy-based interventions to achieve macroscopic structural enhancement. Management of patients undergoing rapid weight reduction with GLP-1 receptor agonists provides a representative clinical example illustrating the rationale for multimodal sequencing, based on clinical observation and expert consensus rather than prospective trial validation. In such individuals, nutritional intervention, exercise programs, and biostimulatory treatments should be initiated concurrently with weight loss rather than deferred until tissue laxity becomes clinically apparent [5]. Prospective clinical studies are needed to validate the efficacy and optimal timing of this proposed sequencing framework.

## Conclusions

Skin longevity medicine is shifting from appearance-oriented anti-aging approaches toward the preservation of tissue function and structural integrity. Future progress should focus on three priorities: establishing clinically applicable functional biomarkers and endpoints beyond visual assessment; advancing the translation of ECM-targeted regenerative strategies, including ECM engineering and biomechanical modulation; and generating high-quality evidence to optimize the sequencing, dosing, and long-term outcomes of multimodal interventions. Emerging technologies such as epigenetic clocks and artificial intelligence may further support personalized assessment and treatment planning, although their clinical implementation requires additional validation.

This review proposes a multimodal intervention pyramid that integrates systemic metabolic and lifestyle optimization, epidermal barrier maintenance, regenerative injectables, and energy-based devices within a unified skin longevity framework. Rather than targeting isolated manifestations of aging, this approach emphasizes the coordinated preservation and restoration of cutaneous structure and function. As functional biomarkers, ECM-based therapies, and multimodal clinical evidence continue to evolve, precision skin longevity management based on individual biological characteristics may become an increasingly practical strategy for extending skin healthspan.

## References

[1] López-Otín C, Blasco MA, Partridge L, Serrano M, Kroemer G (2023). Hallmarks of aging: an expanding universe. Cell.

[2] Kroemer G, Maier AB, Cuervo AM (2025). From geroscience to precision geromedicine: understanding and managing aging. Cell.

[3] Wyles S, Mehta R, Mannick J, Day D (2024). Skin longevity: a paradigm shift in aesthetics. J Cosmet Dermatol.

[4] Wyles SP, Maredia HS, Ansaf RB (2025). Skinspan(TM): a healthy longevity framework for skin aging. Mayo Clin Proc.

[5] Sone N, Goldberg DJ (2026). Longevity in aesthetic dermatology: review of the literature. Dermatol Rev.

[6] Guo J, Fang W, Wang F (2023). Injectable fillers: current status, physicochemical properties, function mechanism, and perspectives. RSC Adv.

[7] Rouch L, De Souto Barreto P, Rolland Y (2026). Accelerated epigenetic and inflammatory aging and intrinsic capacity. JAMA Netw Open.

[8] Zheng J, Wang S, Sun J, Lv J (2026). Dermal fibroblast senescence: the central hub of skin aging-from intrinsic dysfunction to microenvironmental remodeling. Int J Mol Sci.

[9] Haykal D, Flament F, Mora P, Balooch G, Cartier H (2025). Unlocking longevity in aesthetic dermatology: epigenetics, aging, and personalized care. Int J Dermatol.

[10] Yu GT, Ganier C, Allison DB (2025). Mapping epidermal and dermal cellular senescence in human skin aging. Aging Cell.

[11] Naylor EC, Watson RE, Sherratt MJ (2011). Molecular aspects of skin ageing. Maturitas.

[12] Mui BW, Wong JJ, Dumas CE (2026). Hyaluronic acid and tissue mechanics orchestrate mammalian digit tip regeneration. Science.

[13] Haykal D, Flament F, Abdayem R, Lee DH (2026). Toward new clinical evaluation models for skin longevity: the need for predictive and accelerated approaches. Geroscience.

[14] Raynaud E, Lynch B, Ngo B (2026). Daily photoprotection mitigates seasonal skin changes and supports skin longevity: a 1-year in vivo 3D multiphoton microscopy study. Skin Pharmacol Physiol.

[15] Klinngam W, Chaiwichien A, Osotprasit S (2025). Longevity cosmeceuticals as the next frontier in cosmetic innovation: a scientific framework for substantiating product claims. Front Aging.

[16] Wu PY, Hseu JH, Chen YR, Pandey S, Chen SJ, Yang HL, Hseu YC (2026). The skin hydration and anti-ageing benefits of ectoine, achieved through enhanced Src-ERK-mediated HAS-2 and JNK-driven AQP-3 expression in human keratinocytes, along with the inhibition of MMP-1-induced collagen-I degradation in human fibroblasts, both with and without UVB irradiation. Int J Cosmet Sci.

[17] Ke J, Wang J, Wu X, Yan Y (2022). Salidroside ameliorates ultraviolet-induced keratinocyte injury by inducing SIRT1-dependent autophagy. Clin Cosmet Investig Dermatol.

[18] Wang L, Yu D (2026). Nanotechnology-based drug formulation and delivery systems for skin medical aesthetic dermatology: mechanisms, applications, and safety. Front Pharmacol.

[19] Ting W, Chong Y, Long X (2024). A randomized, evaluator-blinded, multicenter study to compare injectable poly-D,L-lactic acid vs hyaluronic acid for nasolabial fold augmentation. Aesthet Surg J.

[20] Maher S, Gold MH (2026). Regulatory, ethical, and safety considerations of exosome-based therapies in dermatology. Dermatol Ther.

[21] Furman D, Auwerx J, Bulteau AL (2025). Skin health and biological aging. Nat Aging.

[22] Haykal D, Flament F, Balooch G (2026). Integrative dermatology for longevity: the synergy of topical and internal approaches. Dermatol Ther (Heidelb).

[23] Asserin J, Lati E, Shioya T, Prawitt J (2015). The effect of oral collagen peptide supplementation on skin moisture and the dermal collagen network: evidence from an ex vivo model and randomized, placebo-controlled clinical trials. J Cosmet Dermatol.

[24] Dakhovnik A, Mantovani M, Knufinke M (2025). A collagen amino acid composition supplementation reduces biological age in humans and increases health and lifespan in vivo. NPJ Aging.

[25] Dolečková I, Kušnierik P, Berka V, Šimek M, Matějková I, Prokopec F, Ovesná P (2025). Oral sodium hyaluronate improves skin hydration, barrier function and signs of aging: a randomized, double-blind, placebo-controlled trial in 150 healthy adults. Sci Rep.

[26] Amin P, Sarabi A, Choe S, Scott S, Suh S, Mesinkovska NA (2025). Oral hyaluronic acid supplement: efficacy in skin hydration, elasticity, and wrinkle depth reduction. J Drugs Dermatol.

[27] Kim SJ, Lee S, Choi YJ (2025). β-Nicotinamide mononucleotide enhances skin barrier function and attenuates UV-B-induced photoaging in mice. Antioxidants (Basel).

[28] Yi KH (2026). Pyrroloquinoline quinone (PQQ) as a mitochondrial rejuvenation strategy in aesthetic dermatology: mechanisms, therapeutic potential, and future clinical applications. Biomolecules.

[29] Hanayama M, Mori K, Ishimoto T, Kato Y, Kawai J (2024). Effects of an ergothioneine-rich Pleurotus sp. on skin moisturizing functions and facial conditions: a randomized, double-blind, placebo-controlled trial. Front Med (Lausanne).

[30] Parga A, Coven H (2025). Traditional diets and skin longevity: Okinawan, Nordic, and Blue Zone insights. J Integr Dermatol.

[31] Nishikori S, Yasuda J, Murata K, Takegaki J, Harada Y, Shirai Y, Fujita S (2023). Resistance training rejuvenates aging skin by reducing circulating inflammatory factors and enhancing dermal extracellular matrices. Sci Rep.

[32] Mortimer T, Zinna VM, Atalay M (2024). The epidermal circadian clock integrates and subverts brain signals to guarantee skin homeostasis. Cell Stem Cell.

[33] Lin L, Chen X, Liu C (2025). Comparative efficacy of topical interventions for facial photoaging: a network meta-analysis. Sci Rep.

[34] Zhao L, Liao Y (2026). The tripartite regulatory framework of the skin extracellular microenvironment and related anti-aging strategies. Mini Rev Med Chem.

[35] Clementi A, Cannarozzo G, Guarino L (2025). Sequential fractional CO(2) and 1540/1570 nm lasers: a narrative review of preclinical and clinical evidence. J Clin Med.

